# The S862C amino acid change in CpMrr1 confers fluconazole resistance in *Candida parapsilosis*

**DOI:** 10.1093/jacamr/dlaf051

**Published:** 2025-04-30

**Authors:** Iacopo Franconi, Noemi Poma, Cosmeri Rizzato, Lorenzo Maltinti, Marco Falcone, Arianna Tavanti, Antonella Lupetti

**Affiliations:** Department of Translational Research and New Technologies in Medicine and Surgery, University of Pisa, Pisa, Italy; SD Mycology Unit, Azienda Ospedaliero-Universitaria Pisana, Pisa, Italy; Department of Biology, University of Pisa, Pisa, Italy; Department of Biology, University of Pisa, Pisa, Italy; Department of Biology, University of Pisa, Pisa, Italy; Department of Clinical and Experimental Medicine, University of Pisa, Pisa, Italy; Infectious Diseases Unit, Azienda Ospedaliero-Universitaria Pisana, Pisa, Italy; Department of Biology, University of Pisa, Pisa, Italy; Department of Translational Research and New Technologies in Medicine and Surgery, University of Pisa, Pisa, Italy; SD Mycology Unit, Azienda Ospedaliero-Universitaria Pisana, Pisa, Italy

## Abstract

**Background:**

*Candida parapsilosis* is an opportunistic pathogen with increasing rates of resistance to fluconazole and voriconazole. Recently, in an outbreak at the Azienda Ospedaliero-Universitaria Pisana, a new amino acid substitution, S862C in the CpMrr1 protein, was found only in azole-resistant strains. The contribution of this mutation to the acquisition of an azole-resistant phenotype was investigated in this study.

**Methods:**

Antifungal resistance in *C. parapsilosis* clinical strains isolated from the outbreak (*n* = 16) was tested by the broth microdilution method and Etest strip. WGS and Sanger sequencing analyses were used for the detection of SNPs. A CRISPR-Cas9–based genome editing strategy was used to induce the C2585G substitution in the *CpMRR1* gene of susceptible *C. parapsilosis* isolates to investigate its role in the acquisition of azole resistance.

**Results:**

The A395T and the newly found C2585G substitution in the *CpMRR1* gene were present in all resistant isolates, but not in the susceptible ones. Such mutations were later induced in the *C. parapsilosis* reference strain ATCC 22019 and in two azole-susceptible clinical isolates in homozygosis, and in heterozygosis only for ATCC 22019 and one azole-susceptible clinical isolate. Both heterozygous and homozygous mutants carrying the C2585G mutation were fluconazole resistant, with some clones also presenting intermediate susceptibility or resistance to voriconazole.

**Conclusions:**

To the best of our knowledge, this is the first study to report the effect on azole resistance of a novel C2585G nucleotide substitution in the *CpMRR1* gene found in clinical isolates recovered during an outbreak of azole-resistant *C. parapsilosis* in a healthcare setting.

## Introduction

Candidiasis is the fourth most common cause of healthcare-associated sepsis with ∼1 565 000 bloodstream infections (BSIs) and 995 000 deaths worldwide per year.^1,2^ Currently, multiple studies have reported an increase in non-*albicans* species among invasive yeast infections.^3,4^ In this scenario, *Candida parapsilosis* has been an opportunistic pathogen causing clinical outbreaks in Europe, Asia, South Africa and Latin America, with worryingly high rates of acquired azole resistance.^4–9^ The WHO included this yeast in the fungal priority list to enhance clinical awareness of such an opportunistic pathogen in order to implement antimicrobial stewardship programmes and infection control measures to prevent its spread among healthcare facilities.^10^

Italy has been the centre of several clinical outbreaks of azole-resistant *C. parapsilosis,*^11–13^ with one currently taking place at Azienda Ospedaliero-Universitaria Pisana, the clinical reference centre of this study, where *C. parapsilosis* has become the most frequently isolated species among all yeasts recovered from BSIs and it is associated with 62.7% and 52.9% of fluconazole and voriconazole resistance, respectively.^14^ The epidemiological survey conducted at Azienda Ospedaliero-Universitaria Pisana indicated that the clinical outbreak started in 2018, when the first resistant strain was isolated, and it peaked in 2021 in terms of prevalence and antifungal resistance rates. The outbreak was still ongoing at the end of this study period in 2024.

Genetic determinants of azole resistance in *Candida* spp. are gain- or loss-of-function mutations that can be located in the transcription factor genes encoding efflux pumps or ergosterol biosynthetic components, or mutations in the *ERG* genes implicated in ergosterol synthesis.^15–17^ In *C. parapsilosis* specifically, the most commonly found resistance mechanism for azole compounds is associated with the presence of the A395T mutation leading to the Y132F amino acid substitution. This is followed by the R398I and the G458S amino acid substitutions in the *CpERG11* gene, involved in the biosynthesis of ergosterol, as well as mutations in the *CpMRR1* (*CPAR2_807270*) gene, encoding a transcription factor regulating the expression of the efflux pump MDR1.^18–20^ However, the R398I substitution has been found only in fluconazole-resistant isolates but has not been associated with resistance. Still, the landscape of antifungal drug resistance in *Candida* spp., and especially in *C. parapsilosis*, is continually evolving, with novel mutations found during new clinical outbreaks.^21^ In light of this, the WHO recommends investigating new clinical outbreaks caused by antimicrobial resistance pathogens using genome sequencing.^22^

As a first infection control measure in response to the clinical outbreak taking place at our hospital, preliminary analyses evaluated the presence of a C2585G substitution in the *CpMRR1* gene that, to the best of our knowledge, has not been previously described. Therefore, the aim of this study was to evaluate the contribution of the C2585G substitution to the development of azole resistance using a clustered regularly interspaced short palindromic repeats (CRISPR)/CRISPR-associated protein 9 (Cas9)–based genome editing strategy.

## Methods

### Ethics

The study was notified (Prot. n. 32634) to the local ethical committee, Comitato Etico di Area Vasta Nord-Ovest, University of Pisa, and conducted in full accordance with the principles of the Declaration of Helsinki. Samples were taken as part of the standard patient care and anonymized by the clinical personnel. Research personnel received and used the strains isolated anonymously. No data regarding patients’ ID and/or personal information were registered or could be retrieved.

### Strains and growth conditions

A set of *C. parapsilosis* isolates (*n* = 16) were kept as frozen stocks at the Mycology Unit, Azienda Ospedaliero-Universitaria Pisana (Table 1). Identification of the isolates was performed by MALDI-TOF (Bruker Daltonics, Bremen, Germany). All *C. parapsilosis* isolates used in this study were grown in yeast/peptone/dextrose (YPD; Panreac Química, S.L.U., Barcelona, Spain) broth or agar plates at 30°C, and kept at −80°C as 40% glycerol-YPD broth frozen stocks. *C. parapsilosis* mutant clones were selected in YPD agar plates supplemented with 100 µg/mL Nourseothricin (YPD-Nou) (Jena Bioscience, Germany).

**Table 1.** Antifungal susceptibility testing by broth microdilution method

			Antifungal agent MIC,^a^ mg/L						
Strain	Clinical ward	Samples	FLC	VRC	ITC	POS	AND	CAS	MYC
CpA	ICU	BSI	64 (R)	0.5 (R)	≤0.0312 (S)	0.0156 (S)	0.0625 (S)	0.0625 (S)	0.125 (S)
CpB	ICU	BSI	64 (R)	0.5 (R)	≤0.0312 (S)	0.0156 (S)	0.25 (S)	0.25 (S)	0.25 (S)
CpC	ICU	BSI	64 (R)	0.5 (R)	0.0625 (S)	0.0156 (S)	0.25 (S)	0.25 (S)	0.25 (S)
CpD	ICU	BSU	64 (R)	0.5 (R)	0.0625 (S)	0.0156 (S)	0.25 (S)	0.25 (S)	0.25 (S)
CpE	ICU	BALF	128 (R)	1 (R)	≤0.0312 (S)	0.0156 (S)	0.25 (S)	0.25 (S)	0.25 (S)
CpF	ICU	BALF	128 (R)	0.5 (R)	≤0.0312 (S)	0.0156 (S)	0.25 (S)	0.25 (S)	0.25 (S)
CpG	ICU	BSI	64 (R)	0.5 (R)	≤0.0312 (S)	0.0156 (S)	0.25 (S)	0.25 (S)	0.25 (S)
CpH^b^	ICU	BSI	64 (R)	0.5 (R)	≤0.0312 (S)	0.0156 (S)	0.25 (S)	0.25 (S)	0.25 (S)
CpI	Surgery	BSI	64 (R)	0.5 (R)	≤0.0312 (S)	0.0156 (S)	0.25 (S)	0.25 (S)	0.25 (S)
CpL	ICU	BSI	1 (S)	0.125 (S)	≤0.0312 (S)	0.0156 (S)	0.25 (S)	0.25 (S)	0.25 (S)
CpM	ICU	BSI	1 (S)	0.125 (S)	≤0.0312 (S)	0.0156 (S)	0.25 (S)	0.25 (S)	0.25 (S)
CpN^b^	ICU	BSI	64 (R)	0.5 (R)	≤0.0312 (S)	0.0156 (S)	0.25 (S)	0.25 (S)	0.25 (S)
CpO	ICU	BSI	64 (R)	0.5 (R)	≤0.0312 (S)	0.0156 (S)	0.125 (S)	0.25 (S)	0.0625 (S)
CpP	ICU	BSI	64 (R)	0.5 (R)	≤0.0312 (S)	0.0156 (S)	0.125 (S)	0.25 (S)	0.0625 (S)
CpQ	ICU	BSI	64 (R)	0.5 (R)	≤0.0312 (S)	0.0156 (S)	0.125 (S)	0.25 (S)	0.0625 (S)
CpR	ID	Urine	1 (S)	0.125 (S)	≤0.0312 (S)	0.0156 (S)	0.25 (S)	0.25 (S)	0.25 (S)
ATCC 22019			1 (S)	0.125 (S)	≤0.0312 (S)	0.0156 (S)	0.25 (S)	0.25 (S)	0.25 (S)

CpA-CpR are clinical strains of *Candida parapsilosis* isolated from the various wards of Pisa University Hospital, collected from different body sites. AND, anidulafungin; BALF,  bronchoalveolar lavage fluid; BSI, bloodstream infection; CAS,  caspofungin; FLC,  fluconazole; ICU, intensive care unit; ID, infectious diseases unit; ITC,  itraconazole; MYC,  micafungin; POS,  posaconazole; VRC,  voriconazole.

^a^S ,  susceptible; R,  resistant, according to EUCAST definitive document E.DEF 7.3.2, 2020.^23^

^b^These two strains were collected from the same patient in two distinct time periods. CpN was isolated 7 days after CpH.

*Escherichia coli* DH5α strain was used for plasmids (pUC57_RIBO_sgADE2 and pSAT1_sgRNA_MRR1_WT) propagation. *E. coli* cells were grown in LB agar and broth (Panreac Química, S.L.U., Barcelona, Spain) at 37°C, supplemented with 100 µg/mL ampicillin (Merck KGaA, Darmstadt, Germany) when necessary. Bacterial stocks were conserved at −80°C in 20% glycerol-LB broth.

### Antifungal susceptibility testing

Antifungal susceptibility testing was performed using the broth microdilution method, according to routine clinical laboratory workflow at Azienda Ospedaliero-Universitaria Pisana, following the recommendations described in the EUCAST definitive document E.DEF 7.3.2.^23^ Briefly, *C. parapsilosis* isolates were plated on Sabouraud solid media supplemented with gentamicin and chloramphenicol (bioMérieux, Craponne, France) and cultured in aerobic conditions at 37°C for 48 h. A homogenized yeast suspension of 0.5 on the McFarland scale was prepared in physiological solution and used to obtain the recommended final inoculum of 0.5 × 10^5^ to 2.5 × 10^5^ cfu/mL. Then 100 µL of the suspension was added in each well of the microtitration plate, Merlin MICRONAUT-AM Antifungal Agents MIC^©^ (Bruker Daltonics GmbH & Co. KG, Germany), to determine the MICs of different antimicrobial agents (amphotericin B, anidulafungin, micafungin, caspofungin, voriconazole, itraconazole and posaconazole). MIC results were read after 48 h of incubation at 37°C and interpreted according to the EUCAST definitive document E.DEF 7.3.2.^23^

The confirmatory Etest strip method MTS^™^ (MIC Test Strip; Liofilchem^®^ S.r.l., Roseto degli Abruzzi, TE, Italy) followed the manufacturer’s instructions (MIC Test Strip Technical Sheet Yeast—MTS24—Rev.6; Liofilchem^®^ S.r.l., Roseto degli Abruzzi, TE, Italy). The solid medium used was RPMI Agar (Liofilchem^®^ S.r.l., Roseto degli Abruzzi, TE, Italy); a 0.5 McFarland suspension in physiological solution was prepared and then streaked onto a plate according to the ‘double-dip’ method. After placing the Etest strip the plate was incubated at 37°C in ambient air and read after 48 h. Interpretation of results followed the EUCAST interpretation criteria definitive document E.DEF 7.3.2.^23^ Fluconazole and voriconazole MIC interpretation was performed according to manufacturers’ instructions. The definition of the inhibition zone was performed through the visualization of the area where a significant decrease in growth density could be observed according to the 80% inhibition principle as reported in the MIC Test Strip Technical Sheet Yeast—MTS24—Rev.6 document. Quality control was assessed with *C. parapsilosis* ATCC 22 019 reference strain following the manufacturers’ instructions under the same conditions as described above (fluconazole MIC range: 1–8 mg/L; voriconazole MIC range: 0.016–0.064 mg/L).

### C. parapsilosis genome sequencing

Genomic DNA extraction from *C. parapsilosis* clinical isolates and the reference strain ATCC 22019 was performed following the protocol described elsewhere.^24^ WGS was run on Illumina NextSeq 500 (Genomix4Life S.r.l., Salerno, Italy). Sequences obtained from every strain were analysed using the Geneious Prime 2022.2 software (https://www.geneious.com), which assembled all reads belonging to the same sample to a reference sequence (*C. parapsilosis* CDC317); SNPs as well as small insertions and deletions were identified. Analysis of the presence of SNPs was restricted to two genes, *CpMRR1* (*CPAR2_807270*) and *CpERG11* (*CPAR2_303740*), associated with the regulation of expression of an efflux pump and ergosterol synthesis, respectively. The presence of SNPs in other genes, such as *TAC1*, *UPC2* and *ERG3*, is known to be associated with azole resistance, and their functional analysis along with the development of mutants is currently ongoing and will be assessed in future studies.

The effect of the identified polymorphisms was assessed *in silico* using the Sorting Intolerant From Tolerant (SIFT; http://sift.jcvi.org) bioinformatics tool.^25^ Visualization of the mutation effect on the amino acid structure of the protein was done using PremPS online software (https://lilab.jysw.suda.edu.cn/research/PremPS/).

Confirmatory Sanger sequencing was performed by Eurofins Genomics (Ebersberg, Germany) on the selected target gene (*CPAR2_807270*). Primers for Sanger sequencing were designed using Primer-BLAST (https://www.ncbi.nlm.nih.gov/tools/primer-blast/index.cgi) and SnapGene software (https://www.snapgene.com/). Analysis and alignments of Sanger sequences were performed with the Unipro Ugene software (https://unipro.ru/; Unipro, Novosibirsk, Russia).^26^ Primers were purchased from the online platform of Merck (Merck KGaA, Darmstadt, Germany). The list of primers used along with primer data and description are shown in Table S1 (available as Supplementary data at *JAC-AMR* Online). *C. parapsilosis* isolates were genotyped by random amplified polymorphic DNA (RAPD) with the primer RPO_2_ (5′-GCGATCCCCA-3′) used to carry out the analysis according to previously published methods.^27^ A phylogenetic tree was created by Dendro-UPGMA online software (http://genomes.urv.cat/UPGMA/).

### Gene editing strategy using the CRISPR-Cas9 system

The CRISPR-Cas9–based system developed for *C. parapsilosis*^28^ was used to evaluate the role of the C2585G nucleotide substitution (S862C amino acid substitution) in the development of azole resistance. A 20 bp guide RNA (gRNA) sequence was designed using the EuPaGDT software (http://grna.ctegd.uga.edu/) based on the WGS data of *C. parapsilosis* ATCC 22019 reference strain. The gRNA sequence was adjacent to a 5′-NGG-3′ protospacer adjacent motif (PAM) in the *CpMRR1* gene region where the mutation of interest (C2585G) was found. Plasmid pUC57_HH_HDV_sgADE2B and the episomal vector pSAT1 were kindly provided by Professor Geraldine Butler, University College Dublin, Dublin. As indicated in the reference protocol,^28^ plasmid pUC57_HH_HDV_sgADE2B was linearized through PCR using Q5 Taq Polymerase (New England Biolabs, MA, USA) with primers GA7 pUC57F and GA7 pUC57R. The selected gRNA for *CpMRR1* was synthesized by primer extension using TaKaRa Ex Premier DNA polymerase (Takara Bio Europe SAS, Saint-Germain-en-Laye, France) with primers CPAR2_807270_C2585G_sgRNA_MRR1_TOP and CPAR2_807270_C2585G_sgRNA_MRR1_BOTTOM (Table S1); such primers contain a 20 bp overlapping region at the 3′ ends. The amplified fragment was then integrated into the linearized pUC57_HH_HDV_sgADE2B plasmid by Gibson assembly (New England Biolabs, MA, USA). The resulting plasmid pUC57_MRR1sgRNA was then used as a template for the amplification of the GAPDH-HH-sgCpMRR1-HDV-t cassette using primers GA_pSAT1_F and GA_pSAT1_R (Table S1). The pSAT1 plasmid, which originally contains a codon-optimized *Cas9* gene and a marker of antifungal resistance, *SAT1*, which confers resistance to nourseothricin, was linearized using the NruI restriction enzyme (New England Biolabs, MA, USA) and the GAPDH-HH-sgCpMRR1-HDV-t cassette was then integrated through Gibson assembly, resulting in the pSAT1_sgMRR1 plasmid, which was then used for the transformation in *C. parapsilosis*.

A DNA repair template (RT-DNA) harbouring the C2585G substitution was designed using *C. parapsilosis* ATCC 22019 strain as reference genome. The RT-DNA was homologous to the target-strand DNA sequence of the *CPAR2_807270* gene; this sequence included 30 nt upstream and 30 nt downstream of the CRISPR-Cas9 cleavage site. Two single nucleotide mutations were included: one with the mutation of interest (C2585G) and a synonymous one in the PAM sequence. The RT-DNA was designed using SnapGene and purchased from Merck. A single-stranded DNA was used as RT-DNA, as it is considered to be less cytotoxic and less prone to random integration in the organism’s genome^29,30^

### C. parapsilosis transformation and mutant screening

The azole-susceptible strains *C. parapsilosis* ATCC 22019, *C. parapsilosis-*L (CpL) and *C. parapsilosis*-M (CpM) (Table 1) were used as parental strains (fluconazole MIC = 1 mg/L for CpL and CpM strains, and MIC = 1.5 mg/L for ATCC 22019 with Etest). Transformation with 5 µg of plasmid pSAT1_sgRNA_MRR1_WT and 5 µg of target-strand ssDNA RT-DNA was performed by electroporation as described elsewhere^31^ (GenePulser; Bio-Rad, Milan, Italy). After electroporation and a 3 h recovery, the yeast suspension was plated on YPD-Nou agar plates and incubated for 48 h at 30°C. Nourseothricin-resistant clones underwent three subculture steps on both YPD agar and YPD-Nou agar, until no growth was observed on YPD-Nou agar plates indicating the plasmid loss. Transformant *C. parapsilosis* clones were then assessed for fluconazole resistance using the E-strip test. Fluconazole-resistant clones were then analysed for the presence of the C2585G mutation by Sanger sequencing of the target gene *CPAR2_807270*.

### Phenotypic characterization of C. parapsilosis mutant clones

Mutant clones and their respective parental strains were characterized phenotypically by testing their growth ability in YPD broth. Growth conditions were set at 30°C, for 24 h incubation in YPD broth. One isolated colony for each mutant and parental strain was cultured overnight at 30°C in 10 mL YPD broth. Then, every strain was diluted in 50 mL YPD broth in order to have the same OD = 0.2 measured at 600 nm and incubated at 30°C. Growth curves were performed assessing the OD of each yeast suspension every 2 h for the first 6 h and every hour after that, up to 24 h. Serial measurements of the OD of the yeast suspension were taken simultaneously using the Ultrospec 10 Cell Density Meter (Amersham Biosciences, Zevenhuizen, The Netherlands). Growth curves were analysed using GraphPad (GraphPad Software, Boston, MA, USA) (https://www.graphpad.com/). The generation times were calculated along the linear range. One-way analysis of variance and *t*-tests were run to compare generation times between mutant clones and respective parental strains.

### Gene expression of CpMRR1

To test if the acquisition of the mutation led to a differential level of *CpMRR1* expression in the C2585G-bearing mutant clones compared with their parental strains, the expression of *CpMRR1* was assessed by RT-PCR. Mutant clones and the parental yeast strains were incubated overnight in 10 mL YPD at 30°C and 200 rpm. The yeast suspension was washed with 1 × Dulbecco’s PBS (Merck KGaA, Darmstadt, Germany), and a 5 × 10^6^ to 5 × 10^7^ cfu/mL suspension was prepared and pelleted at 4500 rpm for 5 min. Total RNA extraction was performed using the Purelink RNA Mini kit (Thermo Fisher Scientific Inc., Waltham, MA, USA). Extracted RNA underwent a purification step using the Turbo DNA-free kit (Thermo Fisher Scientific Inc.). cDNA synthesis was performed using the RevertAid First Strand cDNA Synthesis Kit (Thermo Fisher Scientific Inc.). RT-PCR was performed using the PowerUp SYBR Master Mix (Thermo Fisher Scientific Inc.) and analysed with CFX Connect Real-Time PCR Detection System (Bio-Rad Laboratories, Inc., Hercules, CA, USA). In addition, expression levels of *CpMDR1B* and *CpCDR1B* were also analysed to explore the effect of the C2585G mutation in the *CpMRR1* gene on these target genes. Relative gene expressions of *CpMRR1*, *CpMDR1B* and *CpCDR1B* were quantified using a parental azole-susceptible strain as a control. Primers for RT-PCR can be found in Table S1. The amplification protocol was as follows: 50°C for 2 min, followed by 95°C for 2 min, followed by 39 cycles of 95°C for 15 s and 58°C for 1 min, followed by 65°C for 5 s. Differences in gene expression between mutant clones and parental strains were calculated using the *2^−ΔΔΔCT^* (Livak) method. Expression of the *CpMRR1, CpMDR1B* and *CpCDR1B* genes was normalized using actin as a reference gene. Experiments were performed in three independent replicates.

### Availability of data

Sequence data have been submitted to GeneBank on 31 October 2024. Gene sequences were deposited in GenBank under accession numbers: PQ537842, PQ537843, PQ537844, PQ537845, PQ537846, PQ537847, PQ537848, PQ537849, PQ537850. Sequences will be released one year after deposition.

## Results

### C. parapsilosis azole resistance

All clinical strains, both azole-susceptible and azole-resistant, were collected in 2021 during the clinical outbreak. Antifungal susceptibility profiles of the 16 clinical isolates including the reference strain ATCC 22019 are reported in Table 1. Fluconazole (MIC ≥ 64 mg/L) and voriconazole (MIC ≥ 0.5 mg/L) resistance were detected in 13 clinical isolates out of 16 strains. Two susceptible clinical isolates (CpL and CpM, Table 1) and the reference strain ATCC 22019 were selected as parental strains for the introduction of the C2585G mutation in *CpMRR1* present only in the resistant strains. Other antifungal molecules tested using the broth microdilution test, with their respective MIC values, are reported in Table 1.

### Genome sequencing and RAPD phylogenetic assessment

WGS and Sanger analyses performed on the *CpMRR1* (*CPAR2_807270*) gene of all 17 *C. parapsilosis* strains revealed the presence of the C2585G mutation in heterozygosis in all fluconazole- and voriconazole-resistant isolates. The C2585G mutation leads to the S862C amino acid substitution in the transcription factor CpMrr1, which regulates the expression of both *CpMDR1* and *CpCDR1* genes in *C. parapsilosis*. In addition, at the same genomic analysis, sequencing of the *CpERG11* gene (*CPAR2_303740*) revealed the presence of the A395T mutation, which is associated with the Y132F amino acid substitution in all resistant strains. In summary, all resistant clinical strains harboured both the novel mutation C2585G in the *CpMRR1* and the already known A395T in the *ERG11* genes. Figure S1 shows the phylogenetic tree depicting the 16 isolates categorized into 11 clusters based on the UPGMA method and Dice similarity coefficient. Four of these clusters contained a group of isolates showing a similarity coefficient of one and therefore these isolates can be considered undistinguishable. Whereas some strains clustered separately are still closely related (similarity coefficient ≥0.8), others are more dissimilar leading to the hypothesis that there is more than one strain circulating in the hospital setting.

### Prediction of the C2585G mutation effect on protein structure and function

**Figure 1.**
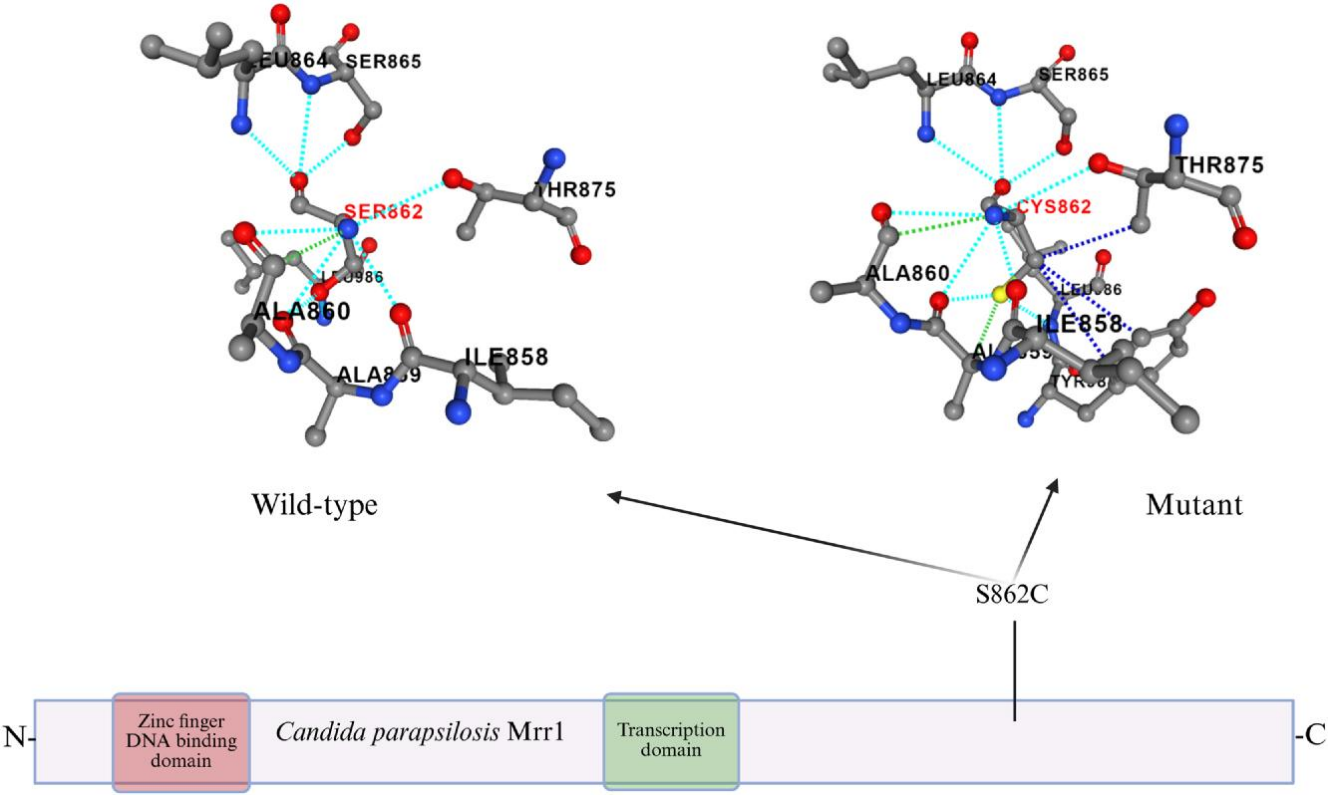
Protein scheme and effect of S862C substitution on the amino acid structure of the protein done with PremPS online software. The amino acid substitution is highlighted in red (SER862, CYS862). Dotted lines represent the interactions between amino acids within the protein structure of both WT (left) and mutant (right) strains. Image created using www.biorender.com.

The C2585G mutation resulted to be non-tolerated (*P* < 0.05) by protein functionality at SIFT prediction (https://sift.bii.a-star.edu.sg/www/SIFT_seq_submit2.html). At the same analysis, the amino acid switch S862C substitution driven by the C2585G mutation was predicted to exert a detrimental effect on protein functionality. Visualization of the effect of the S862C amino acid substitution on the intra-protein interactions is shown in Figure 1.

### Construction of C. parapsilosis mutants carrying the C2585G mutation using CRISPR-Cas9 genome editing

To evaluate the contribution of the C2585G mutation to the development of azole resistance in *C. parapsilosis*, the CRISPR-Cas9 genome editing strategy was applied. Transformation in the azole-susceptible strains *C. parapsilosis* ATCC 22019 and clinical isolates CpM and CpL (which did not harbour the Y132F substitution), resulted in the generation of different transformant clones bearing the C2585G mutation (Table 2).

**Table 2.** Broth microdilution test results and normalized *CpMRR1*, *CpMDR1B* and *CpCDR1B* gene expression ratios of mutant clones compared with parental strains

Clone	*CpMRR1* genotype	Amino acid substitution 862	Antifungal agent MIC,^a^ mg/L				Increased gene expression ratio^b^		
		(*CpMRR1*)	FLC	ITC	VRC	POS	*CpMRR1*	*CpCDR1B*	*CpMDR1B*
ATCC22019	*MRR1*2585C/MRR12585C	S/S	1 (S)	≤0.03125 (S)	0.125 (S)	0.0156 (S)	—	—	—
CpALPHA-4	*MRR1*2585C/MRR12585G	S/C	16 (R)	—	0.25 (I)	0.03125 (S)	2.2	1.7	7.4
CpALPHA-24	*MRR1*2585G/MRR12585G	C/C	64 (R)	—	0.25 (I)	0.03125 (S)	13.5	6.1	15.5
CpM	*MRR1*2585C/MRR12585C	S/S	1 (S)	≤0.03125 (S)	0.125 (S)	0.0156 (S)	—	—	—
CpM-19	*MRR1*2585C/MRR12585G	S/C	8 (R)	—	0.0625 (S)	0.0156 (S)	6.7	2.3	1.44
CpM-WOLV	*MRR1*2585G/MRR12585G	C/C	128 (R)	—	0.5 (R)	0.03125 (S)	37.5	14.7	20
CpM-PHOE	*MRR1*2585G/MRR12585G	C/C	64 (R)	—	0.5 (R)	0.03125 (S)	30.5	5.3	2.5
CpL	*MRR1*2585C/MRR12585C	S/S	1 (S)	≤0.03125 (S)	0.125 (S)	0.0156 (S)	—	—	—
CpL-6	*MRR1*2585G/MRR12585G	C/C	64 (R)	—	0.25 (I)	0.03125 (S)	6.1	3.1	5.7

FLC, fluconazole; ITC, itraconazole; POS, posaconazole; VRC, voriconazole.

^a^S,  susceptible; R,  resistant; I, susceptible increased exposure, according to EUCAST definitive document E.DEF 7.3.2, 2020.^23^

^b^ *2^−ΔΔΔCT^* = normalized expression ratio, Livak method with actin as the reference gene.

In particular, two clones were obtained from *C. parapsilosis* ATCC 22019 (named CpALPHA-4 and CpALPHA-24), three clones were obtained from CpM (named CpM-19, CpM-WOLV, CpM-PHOE) and one clone was obtained from CpL (named CpL-6). Sanger sequencing confirmed the presence of clones containing the C2585G mutation.

### Phenotypic characterization of clones with induced C2585G mutation

**Figure 2.**
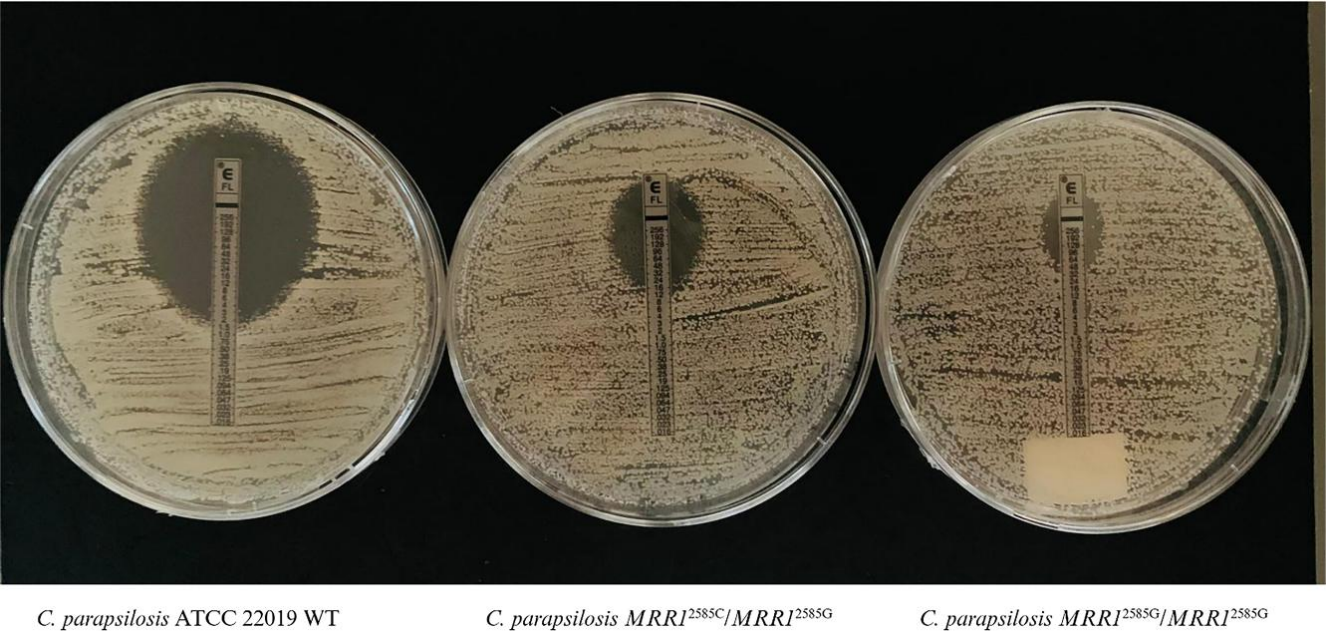
Fluconazole Etest for *Candida parapsilosis* ATCC 22019 reference strain, and heterozygous and homozygous mutant clones for the C2585G substitution in the *CpMRRI* gene. Image created using www.biorender.com.

CpALPHA-4 and CpALPHA-24 clones derived from *C. parapsilosis* ATCC 22019 underwent the Etest susceptibility test for fluconazole showing, respectively, MIC = 16 mg/L and MIC = 48 mg/L (Figure 2). A second confirmatory antifungal susceptibility test was performed on both clones with the broth microdilution assay. CpALPHA-24 presented MIC = 64 mg/L for fluconazole and MIC = 0.25 mg/L (intermediate susceptibility profile) for voriconazole. CpALPHA-4 presented a MIC = 16 mg/L and a MIC = 0.25 mg/L (intermediate susceptibility profile) for voriconazole.

Clones retrieved from susceptible clinical isolates CpM-19, CpM-WOLV, CpM-PHOE and CpL-6 underwent the Etest screening test with fluconazole (MICs: CpM-19 = 16 mg/L; CpM-WOLV > 256 mg/L; CpM-PHOE > 256 mg/L; CpL-6 > 256 mg/L). Secondary confirmation analyses with the broth microdilution method confirmed the resistant phenotypes: fluconazole MIC = 128 mg/L for CpM-WOLV, 64 mg/L for CpM-PHOE and CpL-6, and 8 mg/L for CpM-19; voriconazole MIC = 0.5 mg/L for CpM-WOLV and CpM-PHOE, MIC = 0.25 mg/L for CpL-6, and MIC = 0.03125 mg/L for CpM-19 (Table 2). Results from the broth microdilution test according to the clone and genotype are reported in Table 2.

**Figure 3.**
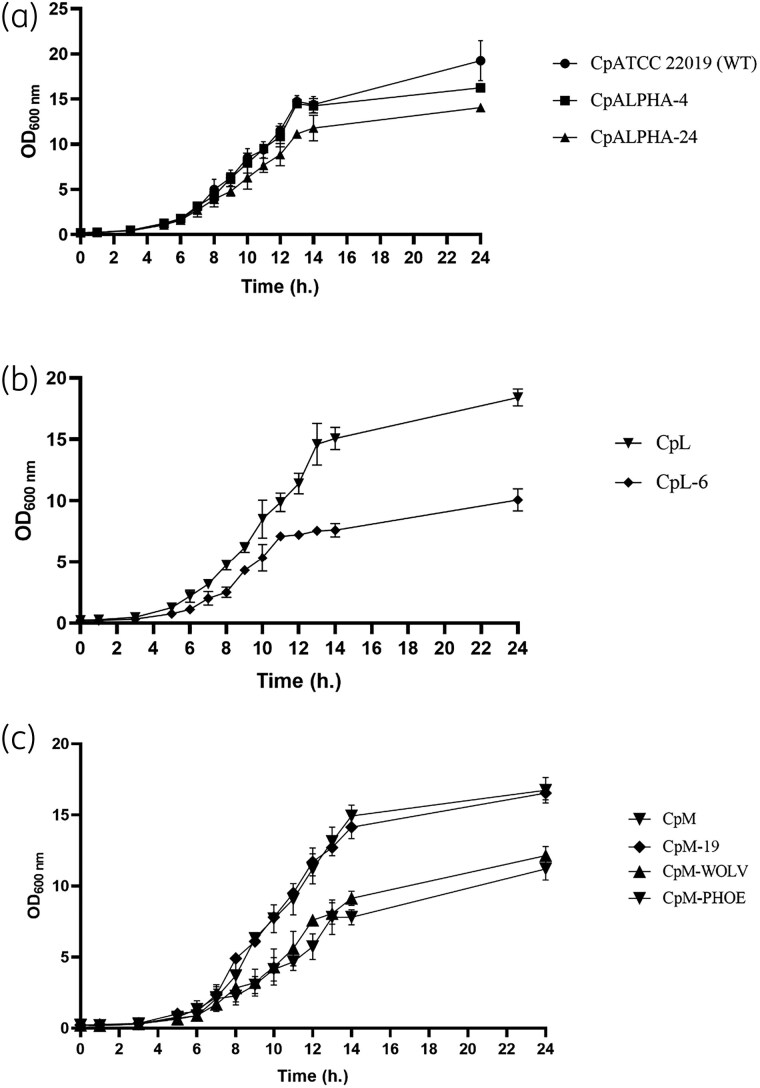
Growth curves for *C. parapsilosis* parental and mutant strains. (a) Parental strains: CpATCC 22019, CpL and CpM. (b) Heterozygous clones: CpALPHA-4 and CpM-19. (c) Homozygous clones: CpALPHA-24, CpL-6, CpM-WOLV and CpM-PHOE. Error bars express SD of the mean.

Growth curves were determined to assess the impact of the C2585G mutation on fungal fitness and growth ability. It was observed that both heterozygous clones (CpALPHA-4, CpM-19) showed similar growth rates to the parental strains (ATCC 22019, CpM), exhibiting similar generation times (Table S2). In contrast, the growth rates of homozygous clones were significantly slower when compared with their respective WT strains (*P* < 0.05; Figure 3 and Table S2).

### Evaluation of CpMRR1, CpMDR1B and CpCDR1B gene expression

The effect of the C2585G mutation on *CpMRR1* expression in *C. parapsilosis* mutant clones was evaluated by RT-PCR. It was observed that both homozygous and heterozygous clones showed an increase in the levels of RNA transcripts compared with parental strains (Table 2). Normalized gene expression levels of *CpMRR1*, *CpMDR1B* and *CpCDR1B* in the homozygous and heterozygous clones compared with parental strains are shown in Table 2. Higher levels of normalized gene expression were found in homozygous clones (CpALPHA-24, CpM-WOLV and CpM-PHOE) compared with heterozygous ones (CpALPHA-4 and CpM-19).

## Discussion

To the best of our knowledge, this is the first study to report the presence of the C2585G mutation (S862C amino acid substitution) in the *CpMRR1* gene of azole-resistant *C. parapsilosis* clinical isolates retrieved during an ongoing clinical outbreak.

Azole-resistant *C. parapsilosis* strains can spread through hand contact from one patient to another, and survival of this yeast within the host can be prolonged, independently of exposure to azole compounds since the fitness costs of azole-resistant strains are similar to those of susceptible ones.^4^ These aspects along with excessive antifungal use are crucial in clinical outbreaks. In this context, where invasive infections caused by drug-resistant clades are increasing, the availability of molecular methods to detect mutations associated with azole resistance may help clinicians save time in choosing the appropriate molecule to treat the invasive form as well as anticipating the resistant phenotype.^32^ This could represent a robust resistance surveillance strategy in the context of antifungal stewardship programmes, leading to the appropriate use of echinocandins when actually needed, thus preventing their overuse and the potential development of tolerance and further resistance.^33^

This study evaluated the contribution of the C2585G nucleotide substitution present in the *CpMRR1* gene (*CPAR2_807270*) to the development of azole resistance in susceptible *C. parapsilosis* strains. Homozygous and heterozygous mutant clones proved to be resistant to fluconazole, and all homozygous clones were also resistant to voriconazole. CpALPHA-4, a heterozygous clone, presented an intermediate susceptibility to voriconazole (MIC = 0.25 mg/L). No effect on other azoles (posaconazole, itraconazole) tested was observed.

The C2585G mutation leads to the S862C substitution in the *CpMRR1* gene, which encodes for the transcription factor of the *CpMDR1B* and *CpCDR1B* genes. Such genes encode members of the MFS (CpMdr1B) and ABC (CpCdr1B) transporter families, respectively, thus implicating an efflux pump-mediated azole resistance in *Candida* spp. The mutation was found in *C. parapsilosis* clinical isolates retrieved during an ongoing outbreak associated with both fluconazole and voriconazole resistance. The genome sequence analyses showed that this newly described mutation was found to be associated with the A395T mutation leading to the Y132F substitution in all azole-resistant clinical isolates. Y132F is the most frequently encountered mutation in azole-resistant *C. parapsilosis* clinical isolates. Such a mutation was not present in the mutant clones and their respective parental isolates used in this study. However, it is known that azole resistance in *Candida* spp. can be the result of the combination of several mutations and phenotypic adaptations. The most common resistance mechanisms in *Candida* spp. are overexpression of efflux pumps, increased ergosterol and chitin synthesis, and/or target modifications of the ergosterol molecule.^5^ Therefore, in this study the contribution of the C2585G mutation to the development of azole resistance was evaluated. SIFT software analysis showed that the C2585G was not tolerated (*P* < 0.05), and visualization of the effect of the mutation on the amino acid structure of the protein using PremPS software confirmed a significant difference in the protein’s structure. However, it is not possible to infer how the amino acid change affects protein function; indeed, SIFT only predicts deleterious effects based on the chemical features/location of the substitution, but does not provide any information as to how this specific change affects this protein.

Studies assessing the relationship between different mutations in the *CpMRR1* gene and the development of azole resistance have not evaluated the precise effect on protein functionality, reporting only overexpression of the mutated gene.^20,34,35^ However, as observed in similar studies conducted on *C. albicans*, mutations in the *CpMRR1* gene can either alter the autoregulatory domain blocking the inhibition of the transcription factor or exert an effect on the DNA-binding domain.^36,37^

The S862C amino acid substitution reported in the present study occurs at the C-terminal domain of the CpMrr1 transcription factor; however, the precise mechanism for the development of resistance is difficult to ascertain since the structure and the function of the different domains in CpMrr1 in *C. parapsilosis* have not yet been fully investigated. Different studies performed on analogous proteins belonging to other species such as *Candida albicans*^37^ and *Saccharomyces cerevisiae*^38^ have shown that mutations in the C-terminal domain of Mrr1p in *C. albicans* might prevent its inhibition by the autoregulatory domain. In *S. cerevisiae* the C-terminal domain is believed to have an activation function for the transcription factor itself. Therefore, despite the lack of consistent background knowledge regarding different domains of the CpMrr1 transcription factor in *C. parapsilosis*, it can be speculated that the novel C2585G mutation found in the resistant clinical isolates could lead to a modification in the autoregulatory domain of CpMrr1, thus inducing activation of both *CpMRR1* and the genes regulated by this transcription factor. It is important to mention also that the novel mutation reported in this study clusters together with other reported amino acid variants previously described in CpMrr1 (Figure 1), such as the A854V substitution investigated by Doorley and colleagues.^20^ Such mutations occurring as homozygotes in the same protein domain have been linked to the development of azole resistance.

The CRISPR-Cas9 single-base editing strategy has proved to be a reliable tool able to induce single point mutations in the *CpMRR1* gene of susceptible *C. parapsilosis* strains, the ATCC 22019 reference strain and two distinct clinical isolates. The presence of the C2585G mutation was confirmed by Sanger sequencing, and it was found to be a mutation associated with fluconazole resistance and intermediate voriconazole susceptibility. Accordingly, gene expression analysis showed an increase in the mRNA levels of the *CpMRR1* gene and in CpMrr1-regulated *CpMDR1B* and *CpCDR1B* genes in all mutant clones, with higher fold-changes in those harbouring the homozygous genotype. These results are in line with previous findings reported by Doorley and colleagues,^20^ who explored the role of other mutations in the same transcription factor–encoding gene in *C. parapsilosis.* Clones bearing the C2585G SNP in homozygosis or heterozygosis were randomly obtained, since no specific differential genome-editing strategy for the controlled generation of both homozygous and heterozygous mutants was used. C2585G homozygous mutant clones were obtained for ATCC 22019, CpM and CpL as parental strains. Heterozygous mutants were instead generated only for ATCC 22019 and CpM.

Regarding the phenotypic characterization, the growth ability of heterozygous clones was similar to their parental strains. However, the homozygous clones showed a reduced growth rate compared with the parental strains. Similar findings have been reported in the study by Hartuis and colleagues,^17^ where a homozygous G1747A mutation (G583R substitution) in the *CpMRR1* gene of *C. parapsilosis* impacted fitness thereby reducing the growth rates of mutated clones. It is also important to mention that the mutation retrieved in our clinical isolates was found to be heterozygous, and the effect on azole resistance of such a genotype was confirmed in both clinical and reference susceptible isolates. This proves that the C2585G mutation leads to the development of azole resistance in clinical isolates.

This study identified and demonstrated for the first time the contribution of the C2585G mutation and the S862C amino acid substitution in the CpMrr1 transcription factor to the development of azole resistance in both reference and clinical *C. parapsilosis* strains. These findings could be beneficial in clinical practice in an infection control prospective. Introducing a newly reported resistance mechanism in the screening procedures of clinical isolates would definitely lead to early detection of new resistant strains, allowing for isolation and disinfection procedures, thus preventing at an early stage the in-hospital spread of such resistant strains.^32^

## Supplementary Material

dlaf051_Supplementary_Data

## Supplementary data

Figure S1 and Tables S1 and S2 are available as Supplementary data at *JAC-AMR* Online.
