# Supplementary material for: The S862C amino acid change in CpMrr1 confers fluconazole resistance in Candida parapsilosis
Source: JAC Antimicrob Resist. 2025 Apr 30;7(3):dlaf051. doi: 10.1093/jacamr/dlaf051 (PMC12041857; doi:10.1093/jacamr/dlaf051)

**Supplementary Table 1**: Primer sequences used in this study

| **Primer Name** | **Sequence (5'-3')** | **Function** |
| --- | --- | --- |
| CPAR2_807270FW | ATGGGAAACAGTGCCGTGAT | Sanger Sequencing |
| CPAR2_807270REV | AACTTTACCCAATCTACGCA | Sanger Sequencing |
| GA7_pUC57F (Lombardi *et al*., 2017)^1^ | TTTAGAGCTAGAAATAGCAAGTTAAAATAAGGCTA GTCC | Linearization of pUC57_HH_HDV_sgADE2B |
| GA7_pUC57R (Lombardi *et* *al*., 2017)^1^ | GAATTTGGAAAAGAAAGAAAGAAAGAAGGAAGG AAGG | Linearization of pUC57_HH_HDV_sgADE2B |
| CPAR2_807270_C2585G_sgRNA_MRR1_TOP | TTCTTTCTTTTCCAAATTCAAGTTCGCTGATGAGTCCGTGAGGACGAAACGAGTAAGCTCGTC | Generation of the *CpMRR1* (*CPAR2_8027270*) sgRNA |
| CPAR2_807270_C2585G_sgRNA_MRR1_BOTTOM | GCTATTTCTAGCTCTAAAACCTGTGTCCAAATTGAGTTCGGACGAGCTTACTCGTTTCGT | Generation of the  *CpMRR1* (*CPAR2_807270*) sgRNA |
| GA_pSAT1_F (Lombardi *et al*., 2017)^1^ | CAGTGAATTGGAGATCGGTACTTCGGTACTTTGGTGTAACTGG | Amplification of the ribozyme cassette |
| GA_pSAT1_R (Lombardi *et al*., 2017)^1^ | GTGTACCGGTATCTCGACGCATTCGCTTTATTTATAAACTCATATACGAAAAATATATAAAAC | Amplification of the ribozyme cassette |
| HDR_DNA_MRR1_MUT | GGTCAGCTGAAGTTTCAATT**GCA**GCTGTG**TGC**AAATTGAGTTCGAGGTATTTTTACGCATG^*^ | Repair Template |
| MRR1CPAR2_807270FW | TCAAGGTGTTTGTCTGATAATG | *CpMRR1* (*CPAR2_807270*) Real time amplification |
| MRR1CPAR2_807270REV | TTGTCAATTTCCATGCGTAA | *CpMRR1* (*CPAR2_807270*) Real time amplification |
| CpMDR1B_F1^2^ (Doorley *et al*. 2022) | CAACTCTAGGAGCAATGACA | *CpMDR1B* (*CPAR2_603010*) Real time amplification |
| CpMDR1B_R1^2^ (Doorley *et al*. 2022) | CGCTGTCGGTGTTGAGAT | *CpMDR1B* *(CPAR2_603010*) Real time amplification |
| CpCDR1B_F1^2^ (Doorley *et al*. 2022) | TGTTCCGCAGTAGGGTCT | *CpCDR1B* (*CPAR2_304370*) Real time amplification |
| CpCDR1B_R1^2^ (Doorley *et al*. 2022) | CCACTTGTGTGCGTTGTAA | *CpCDR1B* (*CPAR2_304370*) Real time amplification |

^*^ The silent mutation in the PAM region is in **bold** and the codon bearing the mutation leading to the S862C amino acid substitution in the CpMrr1 is presented in **bold** and **underlined.**

1. Lombardi L, Turner SA, Zhao F, *et al.* Gene editing in clinical isolates of *Candida parapsilosis* using CRISPR/Cas9. *Sci Rep* 2017; **7**: 8051.

2. Doorley LA, Rybak JM, Berkow EL, *et al*. *Candida* *parapsilosis* Mdr1B and Cdr1B Are Drivers of Mrr1-Mediated Clinical Fluconazole Resistance. *Antimicrobial Agents and Chemotherapy* 2022; 66: e00289-22.

**Supplementary Table 2**: *C. parapsilosis* generation time in YPD broth at 30°C

| **Strains** | **Mean generation time (min)** | **SD** |
| --- | --- | --- |
| ATCC 22019 | 100 | 6 |
| ALPHA4 | 104 | 7 |
| ALPHA24 | 137* | 17 |
| L | 127 | 3 |
| L6 | 182* | 33 |
| M | 101 | 4 |
| M19 | 111 | 14 |
| M-WOL | 186* | 26 |
| M-PHOE | 215* | 55 |

* The generation time (min.) mean value is significantly different with respect to the value calculated for the parental strain P value<0.05.


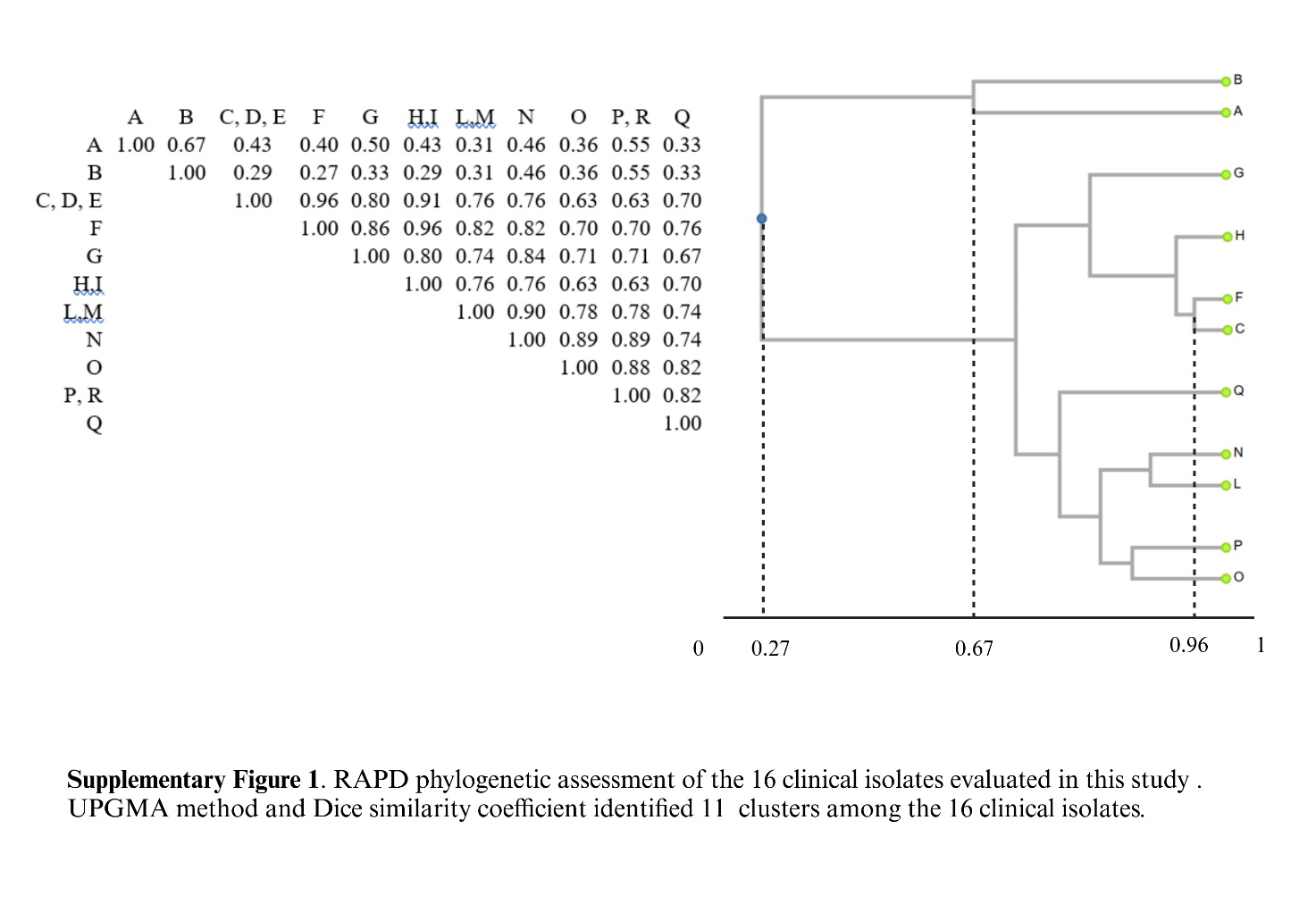

Supplement: dlaf051_Supplementary_Data [file dlaf051_supplementary_data.docx]
